# Supplementary material for: Multi‐dataset identification of innovative feature genes and molecular mechanisms in keratoconus
Source: J Cell Mol Med. 2024 Sep 19;28(18):e70079. doi: 10.1111/jcmm.70079 (PMC11412914; doi:10.1111/jcmm.70079)
Supplement: Supplementary file 2 — Material S2. [file JCMM-28-e70079-s002.zip › 7.GSEA analysis/5.RNF217-AS1/KEGG_PROSTATE_CANCER.html]

Details for gene set KEGG\_PROSTATE\_CANCER[GSEA]

|  || Dataset | RNF217-AS1.group.cls#h\_versus\_l |
| Phenotype | group.cls#h\_versus\_l |
| Upregulated in class | l |
| GeneSet | KEGG\_PROSTATE\_CANCER |
| Enrichment Score (ES) | -0.3443917 |
| Normalized Enrichment Score (NES) | -1.4634136 |
| Nominal p-value | 0.036072146 |
| FDR q-value | 0.28751075 |
| FWER p-Value | 0.808 |
Table: GSEA Results Summary

  

Fig 1: Enrichment plot: KEGG\_PROSTATE\_CANCER      
 Profile of the Running ES Score & Positions of GeneSet Members on the Rank Ordered List

  

| SYMBOL | TITLE | RANK IN GENE LIST | RANK METRIC SCORE | RUNNING ES | CORE ENRICHMENT || 1 | INSRR | na | 1048 | 0.435 | 0.0152 | No |
| 2 | PIK3R5 | na | 1058 | 0.434 | 0.0602 | No |
| 3 | KLK3 | na | 1946 | 0.354 | 0.0715 | No |
| 4 | IKBKB | na | 4368 | 0.238 | 0.0265 | No |
| 5 | BRAF | na | 5517 | 0.199 | 0.0141 | No |
| 6 | PIK3CD | na | 6778 | 0.159 | -0.0056 | No |
| 7 | PDGFB | na | 6818 | 0.158 | 0.0098 | No |
| 8 | IGF1R | na | 6917 | 0.155 | 0.0231 | No |
| 9 | CCNE1 | na | 7161 | 0.148 | 0.0316 | No |
| 10 | TGFA | na | 8488 | 0.116 | 0.0054 | No |
| 11 | E2F3 | na | 8526 | 0.115 | 0.0164 | No |
| 12 | SOS1 | na | 9467 | 0.096 | -0.0007 | No |
| 13 | GSK3B | na | 10962 | 0.071 | -0.0365 | No |
| 14 | PIK3CB | na | 11439 | 0.064 | -0.0436 | No |
| 15 | KRAS | na | 11445 | 0.064 | -0.0371 | No |
| 16 | MTOR | na | 11752 | 0.059 | -0.0398 | No |
| 17 | CREB3 | na | 11909 | 0.057 | -0.0384 | No |
| 18 | PDGFA | na | 12021 | 0.055 | -0.0358 | No |
| 19 | PDPK1 | na | 12622 | 0.047 | -0.0482 | No |
| 20 | TCF7L2 | na | 12940 | 0.043 | -0.0529 | No |
| 21 | IGF1 | na | 13325 | 0.038 | -0.0600 | No |
| 22 | MAPK1 | na | 13362 | 0.038 | -0.0571 | No |
| 23 | PDGFRB | na | 13387 | 0.038 | -0.0539 | No |
| 24 | EGF | na | 13646 | 0.034 | -0.0577 | No |
| 25 | CCNE2 | na | 14005 | 0.031 | -0.0649 | No |
| 26 | PIK3CA | na | 14021 | 0.031 | -0.0621 | No |
| 27 | MAPK3 | na | 14127 | 0.029 | -0.0621 | No |
| 28 | MDM2 | na | 14406 | 0.026 | -0.0674 | No |
| 29 | CREBBP | na | 14557 | 0.024 | -0.0693 | No |
| 30 | CDK2 | na | 14696 | 0.022 | -0.0709 | No |
| 31 | ATF4 | na | 14884 | 0.020 | -0.0742 | No |
| 32 | HSP90AB1 | na | 15515 | 0.014 | -0.0909 | No |
| 33 | AKT2 | na | 15816 | 0.011 | -0.0985 | No |
| 34 | HRAS | na | 15878 | 0.010 | -0.0992 | No |
| 35 | PIK3R1 | na | 16145 | 0.007 | -0.1061 | No |
| 36 | MAP2K2 | na | 16236 | 0.006 | -0.1080 | No |
| 37 | BAD | na | 16311 | 0.006 | -0.1096 | No |
| 38 | CREB1 | na | 16423 | 0.004 | -0.1123 | No |
| 39 | TCF7L1 | na | 16424 | 0.004 | -0.1119 | No |
| 40 | CDKN1B | na | 16572 | 0.003 | -0.1158 | No |
| 41 | CHUK | na | 16613 | 0.003 | -0.1167 | No |
| 42 | CREB3L3 | na | 16617 | 0.003 | -0.1165 | No |
| 43 | HSP90AA1 | na | 16824 | 0.000 | -0.1224 | No |
| 44 | PDGFRA | na | 17252 | -0.004 | -0.1343 | No |
| 45 | NRAS | na | 17304 | -0.005 | -0.1353 | No |
| 46 | ERBB2 | na | 17478 | -0.006 | -0.1396 | No |
| 47 | GSTP1 | na | 18119 | -0.012 | -0.1569 | No |
| 48 | AKT1 | na | 18280 | -0.013 | -0.1601 | No |
| 49 | RB1 | na | 18364 | -0.014 | -0.1610 | No |
| 50 | CCND1 | na | 18820 | -0.019 | -0.1722 | No |
| 51 | RAF1 | na | 18969 | -0.020 | -0.1745 | No |
| 52 | HSP90B1 | na | 19189 | -0.022 | -0.1785 | No |
| 53 | SOS2 | na | 19710 | -0.027 | -0.1907 | No |
| 54 | PTEN | na | 19758 | -0.028 | -0.1891 | No |
| 55 | CTNNB1 | na | 19818 | -0.028 | -0.1879 | No |
| 56 | TCF7 | na | 19939 | -0.029 | -0.1883 | No |
| 57 | CREB3L2 | na | 20073 | -0.031 | -0.1889 | No |
| 58 | INS | na | 20462 | -0.035 | -0.1965 | No |
| 59 | E2F2 | na | 20470 | -0.035 | -0.1931 | No |
| 60 | NFKB1 | na | 20531 | -0.035 | -0.1912 | No |
| 61 | CREB3L1 | na | 20661 | -0.036 | -0.1911 | No |
| 62 | IKBKG | na | 20686 | -0.037 | -0.1880 | No |
| 63 | CASP9 | na | 20912 | -0.039 | -0.1904 | No |
| 64 | RELA | na | 21550 | -0.046 | -0.2040 | No |
| 65 | CREB3L4 | na | 21836 | -0.049 | -0.2071 | No |
| 66 | GRB2 | na | 22374 | -0.055 | -0.2169 | No |
| 67 | FGFR2 | na | 22792 | -0.059 | -0.2228 | No |
| 68 | PIK3R2 | na | 22894 | -0.061 | -0.2194 | No |
| 69 | MAP2K1 | na | 23215 | -0.064 | -0.2219 | No |
| 70 | EP300 | na | 23570 | -0.069 | -0.2249 | No |
| 71 | CDKN1A | na | 24177 | -0.078 | -0.2343 | No |
| 72 | EGFR | na | 24191 | -0.078 | -0.2265 | No |
| 73 | NFKBIA | na | 24662 | -0.085 | -0.2312 | No |
| 74 | ARAF | na | 25299 | -0.095 | -0.2396 | No |
| 75 | SRD5A2 | na | 27543 | -0.138 | -0.2900 | No |
| 76 | FOXO1 | na | 27660 | -0.140 | -0.2787 | No |
| 77 | PDGFC | na | 28424 | -0.160 | -0.2841 | No |
| 78 | PIK3CG | na | 30335 | -0.218 | -0.3165 | No |
| 79 | E2F1 | na | 31302 | -0.254 | -0.3179 | Yes |
| 80 | AKT3 | na | 31832 | -0.281 | -0.3038 | Yes |
| 81 | NKX3-1 | na | 32016 | -0.292 | -0.2786 | Yes |
| 82 | TP53 | na | 32227 | -0.304 | -0.2529 | Yes |
| 83 | AR | na | 32445 | -0.320 | -0.2258 | Yes |
| 84 | CREB5 | na | 32518 | -0.325 | -0.1939 | Yes |
| 85 | PIK3R3 | na | 32895 | -0.352 | -0.1680 | Yes |
| 86 | BCL2 | na | 33060 | -0.365 | -0.1347 | Yes |
| 87 | LEF1 | na | 34024 | -0.487 | -0.1117 | Yes |
| 88 | FGFR1 | na | 34252 | -0.543 | -0.0617 | Yes |
| 89 | PDGFD | na | 34588 | -0.723 | 0.0040 | Yes |
Table: GSEA details [plain text format]

  

Fig 2: KEGG\_PROSTATE\_CANCER      
 Blue-Pink O' Gram in the Space of the Analyzed GeneSet

  

Fig 3: KEGG\_PROSTATE\_CANCER: Random ES distribution      
 Gene set null distribution of ES for **KEGG\_PROSTATE\_CANCER**
